# Stability and Electronic Properties of Mixed Rare-Earth Tri-Metallofullerenes YxDy_3-x_@C_80_ (x = 1 or 2)

**DOI:** 10.3390/molecules29020447

**Published:** 2024-01-16

**Authors:** Yabei Wu, Zhonghao Zhou, Zhiyong Wang

**Affiliations:** 1 Key Laboratory of Advanced Light Conversion Materials and Biophotonics, Department of Chemistry, Renmin University of China, Beijing 100872, China; wyb2019102737@ruc.edu.cn; 2 School of Materials Science and Engineering, Dalian Jiaotong University, Dalian 116028, China

**Keywords:** fullerenes, metallofullerenes, density functional theory calculations

## Abstract

Tri-metallofullerenes, specifically $M_{3}@C_{80}$ where M denotes rare-earth metal elements, are molecules that possess intriguing magnetic properties. Typically, only one metal element is involved in a given tri-metallofullerene molecule. However, mixed tri-metallofullerenes, denoted as M1_x_M2_3-x_@C_80_ (x = 1 or 2, M1 and M2 denote different metal elements), have not been previously discovered. The investigation of such mixed tri-metallofullerenes is of interest due to the potential introduction of distinct properties resulting from the interaction between different metal atoms. This paper presents the preparation and theoretical analysis of mixed rare-earth tri-metallofullerenes, specifically Y_x_Dy_3−x_@C_80_ (x = 1 or 2). Through chemical oxidation of the arc-discharge produced soot, the formation of tri-metallofullerene cations, namely $Y_{2}Dy@C_{80}^{+}$ and ${YDy}_{2}@C_{80}^{+}$ , has been observed. Density functional theory (DFT) calculations have revealed that the tri-metallofullerenes Y_x_Dy_3−x_@C_80_ (x = 1 or 2) exhibit a low oxidation potential, significantly lower than other fullerenes such as C_60_ and C_70_. This low oxidation potential can be attributed to the relatively high energy level of a singly occupied orbital. Additionally, the oxidized species demonstrate a large HOMO-LUMO gap similar to that of Y_x_Dy_3−x_N@C_80_, underscoring their high chemical stability. Theoretical investigations have uncovered the presence of a three-center two-electron metal–metal bond at the center of $Y_{2}DY@C_{80}^{+}$ and ${YDy}_{2}@C_{80}^{+}$ . This unique multi-center bond assists in alleviating the electrostatic repulsion between the metal ions, thereby contributing to the overall stability of the cations. These mixed rare-earth tri-metallofullerenes hold promise as potential candidates for single-molecule magnets.

## 1. Introduction

Fullerenes, which are carbon allotropes characterized by their cage-like structure, exhibit a notable structural feature of possessing a cavity with dimensions smaller than a nanometer. This inherent property allows for the accommodation of metal atoms within the internal space of the fullerene cages, leading to the creation of metallofullerenes. The synthesis, structures, and properties of metallofullerene molecules have undergone thorough examination [1,2,3,4,5,6,7]. The incorporation of metal atoms or clusters enhances the optical, electrical, and magnetic properties of fullerene molecules, offering promising prospects for development in nonlinear optics, single-molecule magnets, fluorescent materials, and various other fields [8,9,10,11,12]. In recent years, researchers have continuously expanded the types and forms of fullerenes by exploring new structures of metallofullerenes, which provides important inspiration for the development of novel molecular-based functional materials.

The internal cavity of a fullerene molecule typically falls within the sub-nanometer scale, capable of accommodating one, two, three, or more atoms [13,14,15,16,17,18,19]. $M@C_{82}$ stands out as the most thoroughly examined mono-metallofullerene [20,21,22,23,24,25,26], primarily attributed to its remarkable production yield. The fullerene structure of $M@C_{82}$ can undergo functionalization, leading to the creation of various derivatives of metallofullerenes. An intriguing cationic mono-metallofullerene is $Li@C_{60}^{+}$ . When compared to C_60_, $Li@C_{60}^{+}$ demonstrates enhanced hydrogenation reactivity [27]. The most-extensively studied di-metallofullerenes are $M_{2}@C_{80}$ [28,29,30]. Theoretical calculations for $M_{2}@C_{80}$ indicate that lanthanide element dimers like $La_{2}$ and $Ce_{2}$ transfer six electrons to the carbon cage, resulting in the electron configuration of $M_{2}^{6+}@C_{80}^{6-}$ . As the metal atom has contributed all its valence electrons, there is no metal–metal bond in $M_{2}@C_{80}$ (M = La, Ce). On the other hand, lanthanide metal dimers, such as $Gd_{2}$ , $Tb_{2}$ , $Dy_{2}$ , and $Er_{2}$ , contribute five electrons to the carbon cage, forming the electron configuration of $M_{2}^{5+}@C_{80}^{5-}$ , while retaining a metal bond occupied by a single electron. The neutral form of $M_{2}@C_{80}$ (M = Gd, Tb, Dy, Er) is unstable due to the open-shell electronic structure of the outer carbon cage. However, covalent derivatization can stabilize these di-metallofullerenes. Of particular interest are the derivatives of $Tb_{2}@C_{80}$ and $Dy_{2}@C_{80}$ , which exhibit unique single-molecule magnet behavior [31].

In the case of metallofullerenes encapsulating three or more metal atoms, it is common to include one or more non-metal atoms within the fullerene cage. Examples include $Sc_{3}N@C_{80}$ , $VSc_{2}N@C_{80}$ , $Sc_{3}C_{2}@C_{80}$ , $Sc_{4}C_{2}@C_{80}$ , $Ti_{3}C_{3}@C_{80}$ , and $Dy_{3}C_{2}@C_{80}$ , etc. [19,32,33,34,35,36]. This is attributed to the significant Coulomb repulsion between the metal ions, which can be alleviated by the presence of non-metal atoms. Besides these cluster fullerenes, several reports have been published on tri-metallofullerenes without non-metal mediators, including $Er_{3}@C_{74}$ , $Y_{3}@C_{80}$ , $Sm_{3}@C_{80}$ , $Tm_{3}@C_{80}$ , and others [37,38,39,40,41,42]. Popov et al. have postulated the presence of a pseudo atom at the core of the $Y_{3}@C_{80}$ molecule, simulating the N atom in $Y_{3}N@C_{80}$ [41]. The interaction between the Y atoms and the pseudo atom mirrors that between the Y atoms and the N atom in $Y_{3}N@C_{80}$ . Notably, the structure of $Sm_{3}@C_{80}$ has been elucidated using single-crystal X-ray diffraction among these tri-metallofullerenes [42]. Recently, our group reported the successful extraction of $Tm_{3}@C_{80}$ from arc-discharge-produced soot through chemical oxidation [43]. Theoretical investigations have suggested the presence of a three-center two-electron metal–metal bonding in these tri-metallofullerenes. Moreover, larger tri-metallofullerenes have been identified in the gas phase via laser ablation [44,45,46,47].

To date, all the previously reported tri-metallofullerenes have exclusively utilized a single category of metal elements. The feasibility of encapsulating diverse metal atoms within the fullerene cage to generate tri-metallofullerenes with mixed elements remains uncertain. This study endeavors to investigate the synthesis of mixed rare-earth tri-metallofullerenes, specifically Y_x_Dy_3−x_@C_80_ (x = 1 or 2). DFT calculations have demonstrated their effectiveness in examining the structures and properties of metallofullerenes, establishing them as a reliable and robust method [10]. In this work, we assess the stability and electronic characteristics of Y_x_Dy_3−x_@C_80_ (x = 1 or 2) through DFT calculations.

## 2. Results and Discussion

**Figure 1:**
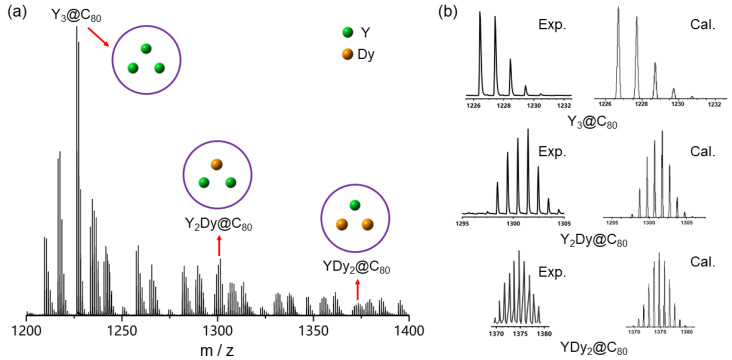
(**a**) Mass spectrum for the oxidized metallofullerenes. The signals from Y_3_@C_80_, Y_2_Dy@C_80_ and YDy_2_@C_80_ are observed. (**b**) The experimental and calculated isotope distributions of the samples.

Following the arc-discharge process using a Y and Dy precursor mixture (with a molar ratio of Y:Dy = 2:1), we conducted the chemical oxidation of the resulting soot. Figure 1 displays the mass spectrum of the oxidized products. The most prominent peak corresponds to $Y_{3}@C_{80}$ . Signals indicative of mixed rare-earth tri-metallofullerenes, specifically $Y_{2}Dy@C_{80}$ and ${YDy}_{2}@C_{80}$ , were also observed. This marks the first observation of the existence of mixed rare-earth tri-metallofullerenes. In this work, the mass spectra were measured in the positive mode. Typically, the peak intensity in the mass spectra may not accurately represent the yields of different metallofullerenes due to their varying ionization energies. However, in this study, the trimetallofullerenes ( $Y_{3}@C_{80}$ , Y_2_Dy@C_80_ and YDy_2_@C_80_) have very similar ionization energies, with calculations yielding values of 5.15, 5.14, and 5.13 eV, respectively. Consequently, based on the intensity of the mass peaks, the yields decrease in the following order: $Y_{3}@C_{80}$ > Y_2_Dy@C_80_ > YDy_2_@C_80_. Other peaks in the mass spectrum correspond to mono-metallofullerens M_2_@C_2n_ and di-metallofullerenes $M_{2}@C_{2n}$ (M = Y or Dy). Fullerenes C_60_ and C_70_ were also produced in the arc-discharge process. However, their solubility in dichloromethane—the solvent used—is low. On the other hand, oxidized metallofullerenes readily dissolve in dichloromethane. As a result, the metallofullerenes were concentrated in the extract.

**Figure 2:**
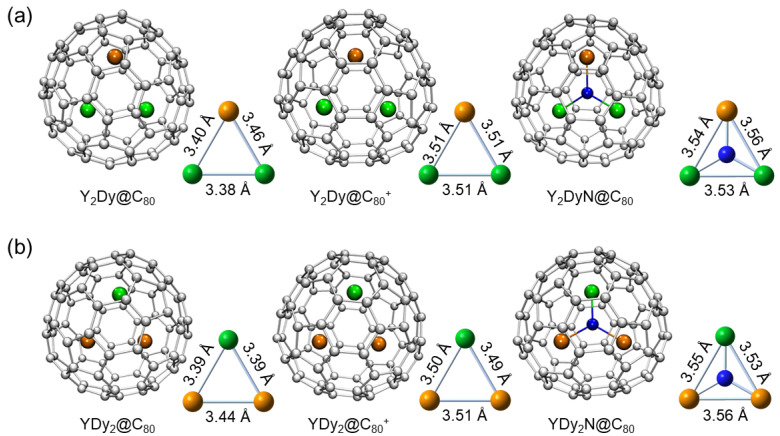
The optimized structures of (**a**) Y_2_Dy@C_80_, $Y_{2}Dy@C_{80}^{+}$ , Y_2_DyN@C_80_ and (**b**) YDy_2_@C_80_, ${YDy}_{2}@C_{80}^{+}$ , YDy_2_N@C_80_. The metal–metal distances are also shown. The C, N, Y, and Dy atoms are denoted by gray, blue, green, and brown colors, respectively.

Previous investigations have suggested that both $Y_{3}@C_{80}$ and $Tm_{3}@C_{80}$ share the same fullerene cage as $Y_{3}N@C_{80}$ and $Tm_{3}N@C_{80}$ , specifically the $I_{h}$ -symmetric $C_{80}$ cage [41,43]. It should be noted that the symmetry of this fullerene is lowered to $C_{3V}$ due to the Jahn–Teller symmetry reduction. The cage obtains its highest symmetry only when filled with metals [48]. To ascertain whether Y_2_Dy@C_80_ and YDy_2_@C_80_ also adopt this particular fullerene cage, we conducted DFT calculations. Prior to this study, Popov et al. performed theoretical calculations to establish the energy order of $C_{80}^{6-}$ isomers [49]. We considered the nine most stable $C_{80}^{6-}$ isomers as potential cages for encapsulating the Y and Dy atoms, forming Y_2_Dy@C_80_ and YDy_2_@C_80_. Consequently, we obtained nine isomers for Y_2_Dy@C_80_ and YDy_2_@C_80_. The relative energies, computed using the PBE0 functional, are presented in Table 1 and Table 2. Our analysis reveals that the *I_h_*-symmetric cage corresponds to the lowest energy isomer for both Y_2_Dy@C_80_ and YDy_2_@C_80_. Their molecular structures are depicted in Figure 2.

When encapsulating multiple metal atoms within a fullerene cage, it is often necessary to introduce one or two non-metal atoms between them to counteract the strong Coulomb repulsion. Examples of this phenomenon include $Sc_{3}C_{2}@C_{80}$ , $Dy_{3}C_{2}@C_{80}$ , and other cases [33,36,50]. Theoretical confirmation exists showing that certain metal carbide cluster fullerenes, such as M_x_C_y_@C_2n_, possess lower energy compared to their metallofullerene counterparts M_x_@C_2n+y_. To assess the stability of Y_2_Dy@I_h_−C_80_ (referred to as Y_2_Dy@C_80_ hereafter) and Y_2_DyC_2_@C_78_, we also conducted energy calculations for Y_2_DyC_2_@C_78_. The computational results indicate that Y_2_DyC_2_@C_78_ exhibits higher energy than Y_2_Dy@C_80_, as detailed in Table 3. Consequently, it can be concluded that Y_2_Dy@C_80_ is thermodynamically more stable than its metallic carbon cluster counterpart, Y_2_DyC_2_@C_78_. A similar conclusion was drawn for YDy_2_@C_80_ (Table 4).

The stability of Y_2_Dy@C_80_ and ${YDy}_{2}@C_{80}$ was assessed by computing the binding energies ( $\Delta E_{b}$ ) between the metal cluster and fullerene cage using hypothetical reactions (1) and (2). The definition of binding energy is as follows: $\Delta E_{b}$ = E(fullerene) + E(metal cluster) − E(metallofullerene). Higher binding energy values indicate a greater stability of metallofullerenes. The binding energy calculated for Y_2_Dy_2_@C_80_ is 281 kcal/mol. On the other hand, the binding energy calculated for YDy_2_@C_80_ is smaller, specifically 273 kcal/mol. The computed binding energy for Y_3_@C_80_ is 289 kcal/mol. This sequence of the binding energy for Y_3_@C_80_, Y_2_Dy@C_80_, and YDy_2_@C_80_ aligns with the order of their peak intensities in the mass spectrum. Consequently, it is reasonable to infer that the yield of tri-metallofullerenes is correlated with the binding energy. We have computed the van der Waals volume, which refers to the space enclosed within the isosurface with an electron density of 0.001 a.u., for C_80_, Y_3_@C_80_, Y_2_Dy@C_80_, and YDy_2_@C_80_. The obtained values are 820, 870, 875, and 876 Å^3^ for C_80_, Y_3_@C_80_, Y_2_Dy@C_80_, and YDy_2_@C_80_, respectively. It is evident that the volume expands due to the incorporation of the metal cluster. The ionic radius of Dy^3+^ (0.091 nm) is larger than that of Y^3+^ (0.089 nm). According to the computed binding energies, it can be inferred that the Y_2_Dy cluster is better suited than the YDy_2_ cluster for encapsulation within the C_80_ cage.

For comparison, we also calculated the binding energy for Y_2_DyN@C_80_ and YDy_2_N@C_80_, both of which are nitride clusterfullerenes known for their exceptional stability (reactions (3) and (4)). Previous studies have examined the stability of the nitride clusterfullerene M_3_N@C_80_ for different metal atoms. These studies have revealed that certain rare-earth elements, such as La and Nd, are too large to fit within the *I_h_*-C_80_ fullerene cage, while Dy and Y atoms possess suitable sizes. The computed binding energies for La_3_@C_80_ and Nd_3_@C_80_ are 217 and 237 kcal/mol, respectively, which are significantly lower than those of Y_3_@C_80_, Y_2_Dy@C_80_, and YDy_2_@C_80_. Signals corresponding to La_3_@C_80_ and Nd_3_@C_80_ were not detected in the oxidized products. We attribute this observation to the substantial size of the La and Nd atoms. Our calculations demonstrate that the binding energies of Y_2_DyN@C_80_ and YDy_2_N@C_80_ are 269 and 257 kcal/mol, respectively. The binding energies of Y_2_Dy@C_80_ and YDy_2_@C_80_ are higher than those of Y_2_DyN@C_80_ and YDy_2_N@C_80_, indicating that Y_2_Dy@C_80_ and YDy_2_@C_80_ exhibit greater stability. It is important to note that the arc-discharge synthesis method involves conditions that are far from equilibrium. These conditions, referred to as non-equilibrium plasma by Osawa [51], prioritize structural and flux parameters over energy parameters. In previous studies, auxiliary parameters, such as activation energies and stochastic descriptors, have been utilized to predict the formation of fullerenes [52]. In the case of Y_3_@C_80_, Y_2_Dy@C_80_, and YDy_2_@C_80_, the analysis based on stochastic descriptors may offer valuable insights. This aspect will be explored in our future research.

(1) $$\begin{array}{ccc} Y_{2}\mathrm{Dy}+C_{80} & ⟶Y_{2}\mathrm{Dy}@C_{80} & \Delta E_{b}\left(1\right)=281\;\mathrm{kcal}/\mathrm{mol} \end{array}$$

(2) $$\begin{array}{ccc} {\mathrm{YDy}}_{2}+C_{80} & ⟶{\mathrm{YDy}}_{2}@C_{80} & \Delta E_{b}\left(2\right)=273\;\mathrm{kcal}/\mathrm{mol} \end{array}$$

(3) $$\begin{array}{ccc} Y_{2}\mathrm{DyN}+C_{80} & ⟶Y_{2}\mathrm{DyN}@C_{80} & \Delta E_{b}\left(3\right)=269\;\mathrm{kcal}/\mathrm{mol} \end{array}$$

(4) $$\begin{array}{ccc} {\mathrm{YDy}}_{2}N+C_{80} & ⟶{\mathrm{YDy}}_{2}N@C_{80} & \Delta E_{b}\left(4\right)=257\;\mathrm{kcal}/\mathrm{mol}. \end{array}$$

Chemical oxidation plays a crucial role in the extraction of Y_2_Dy@C_80_ and YDy_2_@C_80_. Attempting direct extraction using conventional fullerene solvents such as toluene and CS_2_ proved unsuccessful for these mixed rare-earth tri-metallofullerenes. This scenario echoes our earlier findings with Tm_3_@C_80_ [43]. The inference drawn is that Y_2_Dy@C_80_ and YDy_2_@C_80_ tend to undergo oxidation, and their resulting cations demonstrate chemical stability. Subsequently, we computed the ionization energy of Y_2_Dy@C_80_ and YDy_2_@C_80_, and observed that they exhibit relatively low ionization energy values. Specifically, the ionization energy of Y_2_Dy@C_80_ is 5.49 eV, while that of YDy_2_@C_80_ is 5.47 eV. These values are significantly smaller than the calculated ionization energies of C_60_ (7.76 eV) and C_70_ (7.56 eV). It has been reported that Li@C_60_ can undergo oxidation to form $Li@C_{60}^{+}$ , leading to the creation of various stable ionic compounds [53,54,55]. Notably, the calculated ionization energy of Y_2_Dy@C_80_ and YDy_2_@C_80_ is comparable to that of Li@C_60_ (5.73 eV), computed at the same level. Consequently, it can be inferred that Y_2_Dy@C_80_ and YDy_2_@C_80_ molecules may undergo chemical oxidation to produce their respective cations, namely $Y_{2}Dy@C_{80}^{+}$ and ${YDy}_{2}@C_{80}^{+}$ . Figure 2 presents the DFT-optimized structures of $Y_{2}Dy@C_{80}^{+}$ and ${YDy}_{2}@C_{80}^{+}$ . For comparison, the DFT-optimized structures of Y_2_DyN@C_80_ and YDy_2_N@C_80_ are also depicted in Figure 2. The comparison reveals that the structures of $Y_{2}Dy@C_{80}^{+}$ and ${YDy}_{2}@C_{80}^{+}$ closely resemble those of Y_2_DyN@C_80_ and YDy_2_N@C_80_, respectively. The metal–metal distances are around 3.5 angstrom in these cations and neutral molecules.

**Figure 3:**
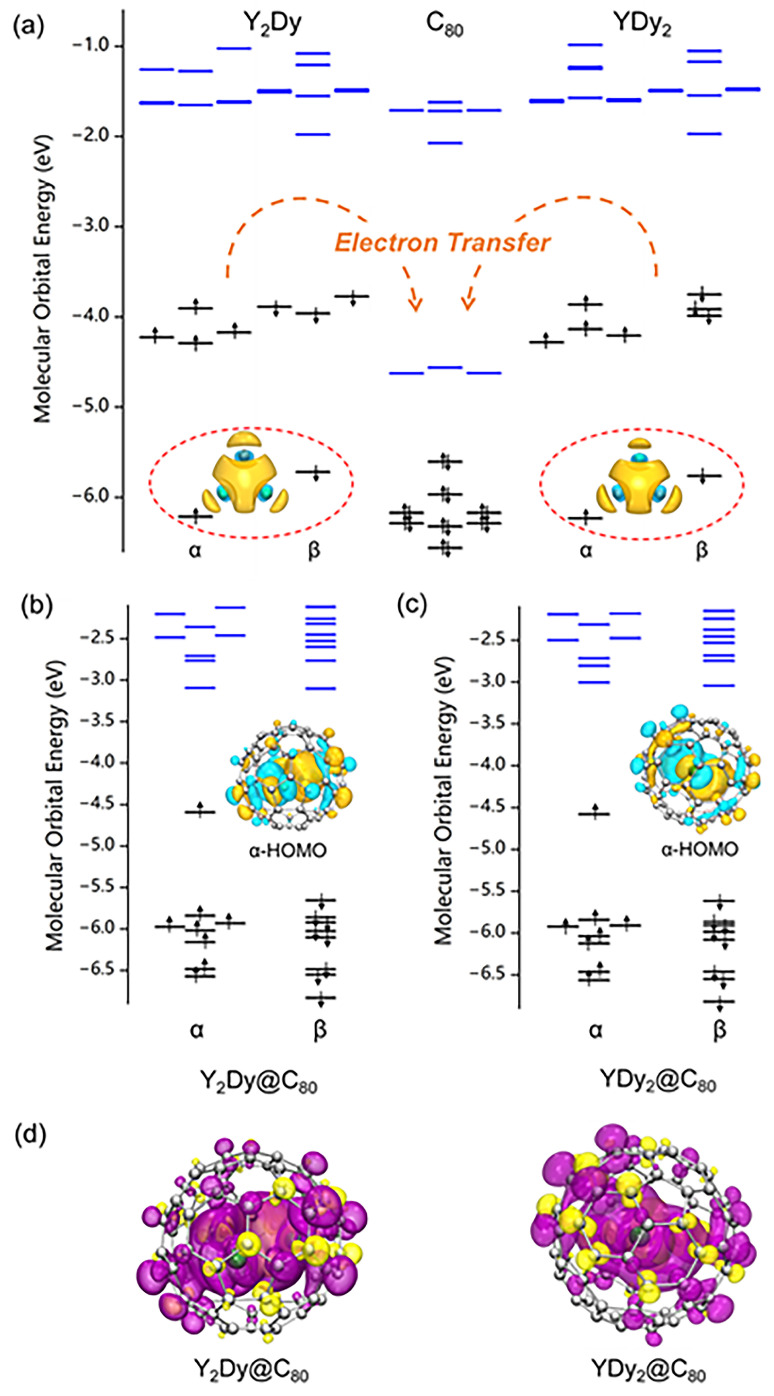
(**a**) The calculated molecular orbital energy level of C_80_ compared to that of Y_2_Dy and YDy_2_ clusters. The molecular orbitals corresponding to three-center two-electron metal–metal bonding in the clusters are encircled with dotted lines. The spatial distribution of the three-center two-electron metal–metal bonding orbital for the Y_2_Dy and YDy_2_ clusters is shown. (**b**,**c**) The molecular orbital energy diagrams for Y_2_Dy@C_80_ and YDy_2_@C_80_. (**d**) The calculated spin density distribution for Y_2_Dy_2_@C_80_ and YDy_2_@C_80_ (isovalue = 0.0004).

The low ionization energy exhibited by Y_2_Dy@C_80_ and YDy_2_@C_80_ can be elucidated by examining the energy of molecular orbitals. We carried out calculations on the clusters of Y_2_Dy, YDy_2_, and the fullerene C_80_. Figure 3a displays the calculated molecular orbital diagrams. The LUMO/LUMO+1/LUMO+2 orbitals of the C_80_ molecule are nearly degenerate, while there is a significantly large energy gap between LUMO + 2 and LUMO + 3. As a result, the C_80_ cage has a preference to accept six electrons in order to achieve a stable electronic configuration. In the case of the Y_2_Dy, YDy_2_ clusters, there are seven electrons with relatively high energy levels in the frontier molecular. Upon encapsulation of the metal cluster into the C_80_ cage, six electrons are transferred to the LUMO/LUMO + 1/LUMO + 2 orbitals of C_80_, leaving one unpaired electron on the metal cluster. In the case of the bare Y_2_Dy and YDy_2_ clusters, there exist molecular orbitals that are associated with three-center two-electron bonding (encircled with dotted lines in Figure 3a). These specific molecular orbitals retain their bonding characteristics when the metal cluster is enclosed within C_80_ due to their comparably low energy levels.

The bond lengths for Dy-Y(1), Dy-Y(2), and Y(1)-Y(2) in bare Y_2_Dy cluster are 3.21, 3.21, and 3.18 angstrom, respectively, which are much shorter than those in the metallofullerene Y_2_Dy@C_80_. We carried out a Wisberg bond order analysis for Y_2_Dy, YDy_2_, Y_2_Dy@C_80_ and YDy_2_@C_80_. In Y_2_Dy, the bond orders for Dy-Y(1), Dy-Y(2), and Y(1)-Y(2) are 1.76, 1.76, and 2.05, respectively, indicating a strong bonding interaction between the metal atoms. However, in Y_2_Dy@C_80_, the bond orders for Dy-Y(1), Dy-Y(2), and Y(1)-Y(2) decrease to 0.56, 0.62, and 0.75, respectively. This weakening of the metal atom bonding is a result of electron transfer from the metal cluster to the fullerene cage. At the same time, the repulsion between the metal ions becomes stronger in the metallofullerene compared to the bare metal cluster. A similar trend is observed for YDy_2_ and YDy_2_@C_80_. In YDy_2_, the bond orders for Dy(1)-Dy(2), Dy(1)-Y, and Dy(2)-Y are 1.64, 1.94, and 1.94, respectively. These bond orders decrease to 0.54, 0.69, and 0.67 in YDy_2_@C_80_.

Figure 3b,c portray the calculated molecular orbitals for Y_2_Dy@C_80_ and YDy_2_@C_80_. In both instances, the HOMO for the alpha spin possesses a relatively high energy level. Figure 3d displays the calculated spin density distribution for Y_2_Dy@C_80_ and YDy_2_@C_80_. They exhibit a spatial distribution that is comparable to the alpha HOMO, signifying that the alpha HOMO primarily corresponds to the unpaired electron in the molecule. The high energy level of the alpha HOMO makes the molecule prone to oxidation into a cation. Upon careful analysis of the alpha HOMO’s shape, it has been determined that it is a bonding orbital. The removal of an electron from this orbital would result in a reduction of the bonding strength between the metal atoms. Consequently, the cations $Y_{2}Dy@C_{80}^{+}$ and ${YDy}_{2}@C_{80}^{+}$ exhibit longer metal–metal distances compared to the neutral molecules Y_2_Dy@C_80_ and YDy_2_@C_80_ (Figure 2).

**Figure 4:**
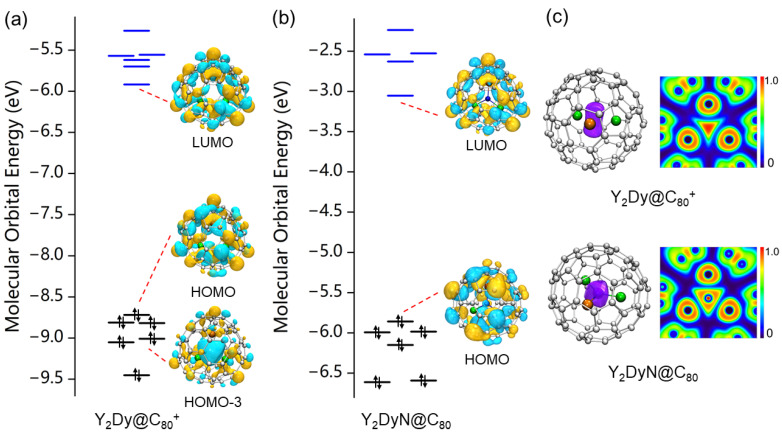
The molecular orbital energy diagrams for (**a**) Y_2_Dy@ $C_{80}^{+}$ and (**b**) Y_2_DyN@C_80_. HOMO-3 in (**a**) corresponds to a three-center σ bond. (**c**) Three-dimensional ELF isosurface (**left**, isovalue = 0.6) and color-filled two-dimensional ELF maps at the metal cluster plane (**right**) for Y_2_Dy@ $C_{80}^{+}$ and Y_2_DyN@C_80_.

As previously mentioned, the molecular structures of $Y_{2}Dy@C_{80}^{+}$ and ${YDy}_{2}@C_{80}^{+}$ closely mirror those of Y_2_DyN@C_80_ and YDy_2_N@C_80_, respectively. The metal–metal distances in $Y_{2}Dy@C_{80}^{+}$ and ${YDy}_{2}@C_{80}^{+}$ closely resemble those in Y_2_DyN@C_80_ and YDy_2_N@C_80_. Our calculations further reveal that they share similar electronic structures. Figure 4a,b illustrate the molecular orbital energy diagrams for $Y_{2}Dy@C_{80}^{+}$ and Y_2_DyN@C_80_. Notably, the HOMO and LUMO of $Y_{2}Dy@C_{80}^{+}$ exhibit strikingly similar shapes to that of Y_2_DyN@C_80_. Meanwhile, the calculated HOMO-LUMO gap of $Y_{2}Dy@C_{80}^{+}$ (2.80 eV) is identical to that of Y_2_DyN@C_80_ (2.80 eV). Among all the reported metallofullerenes, M_3_N@C_80_ represents a type with a notably large HOMO-LUMO gap. DFT calculations in this study unveil that $Y_{2}Dy@C_{80}^{+}$ also possesses a substantial HOMO-LUMO gap, suggesting high stability for this cation. It is noteworthy that the neutral Y_2_Dy@C_80_ exhibits a relatively small HOMO-LUMO gap (1.47 eV using the PBE0 functional, and 0.30 eV using the PBE functional), indicating its chemical reactivity. The significant increase in the HOMO-LUMO gap for $Y_{2}Dy@C_{80}^{+}$ enhances the chemical stability of Y_2_Dy@C_80_ upon oxidation.

**Figure 5:**
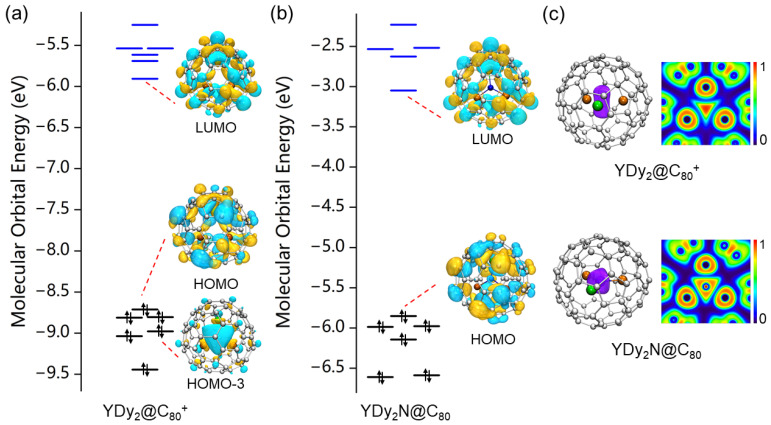
The molecular orbital energy diagrams for (**a**) ${YDy}_{2}@C_{80}^{+}$ and (**b**) YDy_2_N@C_80_. HOMO-3 in (**a**) corresponds to a three-center σ bond. (**c**) Three-dimensional ELF isosurface (**left**, isovalue = 0.6) and color-filled two-dimensional ELF maps at the metal cluster plane (**right**) for YDy_2_@ $C_{80}^{+}$ and YDy_2_N@C_80_.

It is noteworthy that the ELF distribution of $Y_{2}Dy@C_{80}^{+}$ closely resembles that of Y_2_DyN@C_80_ (Figure 4c). In the case of $Y_{2}Dy@C_{80}^{+}$ , the ELF distribution at the molecular center indicates the presence of covalent bonding among the three metal atoms, specifically forming a three-center metal–metal bond. This bond corresponds to the HOMO-3 molecular orbital, as depicted in Figure 4a. For Y_2_DyN@C_80_, where the formal charge of the metal atom is +3, strong electrostatic repulsion exists between the metal ions. The central N^3−^ ion acts as a mediator, mitigating the repulsion between the metal ions. In the bare Y_2_Dy cluster, the bond lengths for Dy-Y(1), Dy-Y(2), and Y(1)-Y(2) are 3.21, 3.21, and 3.18 angstrom, respectively. However, in $Y_{2}Dy@C_{80}^{+}$ , the bond lengths for Dy-Y(1), Dy-Y(2), and Y(1)-Y(2) increase to 3.51, 3.51, and 3.51 angstrom, respectively, indicating strong repulsion between these metal ions. A similar trend is observed for YDy_2_ and YDy_2_@C_80_. In YDy_2_, the bond lengths for Dy(1)-Dy(2), Dy(1)-Y, and Dy(2)-Y are 3.24, 3.21, and 3.21 angstrom, respectively. These bond lengths increase to 3.51, 3.49, and 3.50 angstrom in ${YDy}_{2}@C_{80}^{+}$ . In the case of $Y_{2}Dy@C_{80}^{+}$ , the three-center σ bond serves a role akin to that of the N^3−^ ion in Y_2_DyN@C_80_. The three-center σ bond can mitigate the repulsion between the metal ions and allows three metal atoms to be accommodated within the C_80_ cage without the need for a nonmetal mediator. Thus, the three-center σ bond emerges as a crucial factor contributing to the stabilization of the tri-metallofullerene cation. Furthermore, our calculations indicate that the electronic structure of ${YDy}_{2}@C_{80}^{+}$ closely mirrors that of YDy_2_N@C_80_. They exhibit analogous frontier molecular orbitals and comparable ELF distribution at the molecular center (Figure 5). Consequently, the stability of ${YDy}_{2}@C_{80}^{+}$ can be elucidated in a manner similar to that of $Y_{2}Dy@C_{80}^{+}$ .

The magnetic characteristics of rare-earth metallofullerenes are intriguing. Some of them are formed in high spin states [56,57]. There have been reports highlighting the single-molecule magnet behavior of the clusterfullerenes Y_2_DyN@C_80_ and YDy_2_N@C_80_ [58]. Given that the cations $Y_{2}Dy@C_{80}^{+}$ and ${YDy}_{2}@C_{80}^{+}$ share similar geometrical and electronic structures with Y_2_DyN@C_80_ and YDy_2_N@C_80_, respectively, these cations emerge as promising candidates for single-molecule magnets. Exploring the separation of these cations in future studies would constitute valuable and important research.

## 3. Experimental and Theoretical Methods

The metallofullerenes were synthesized using the arc-discharge technique. The anode was made of a carbon rod, while the cathode was a hollow carbon rod filled with a mixture of Y_2_O_3_, Dy_2_O_3_ (molar ratio of Y:Dy = 2:1), and graphite powders. Prior to the discharge, the cathode was heated by connecting it to the anode and applying a current of 120 A for an hour under a dynamic vacuum. The actual arc-discharge process occurred at a current of 120 A in a 200 Torr helium environment, with a distance of approximately 1 cm between the anode and cathode. The raw soot generated from the arc was subjected to oxidation using AgSbF_6_ in dichloromethane within a nitrogen-filled glove box for a duration of 24 h. Following the oxidation process, the solution was separated from the insoluble soot residue through centrifugation and filtration. No chromatographic purification was carried out. The sample was then analyzed using a mass spectrometer (AB SCIEX 5800 MALDI TOF/TOF, Toronto, Canada) to obtain a mass spectrum, with the analysis conducted in the positive mode without using any matrix.

We carried out DFT calculations to examine the structure and properties of the metallofullerenes. To optimize the structure and determine the molecule’s energy, the PBE0 functional [59] was employed. The carbon and nitrogen atoms were treated using the 6-31G(d) basis set [60]. The Y and Dy atoms were considered using the SDD pseudopotentials and the corresponding basis sets. The 6-311G(d) basis set was used for single-point energy calculations [61]. To investigate the bonding properties of the molecules, the MULTIWFN program [62] was utilized to perform electron localization function (ELF) analysis [63] and Wiberg bond order analysis on a Lowdin orthogonalized basis. All DFT calculations were conducted using Gaussian16 version A03 [64]. The visualization of the calculation results was accomplished using the Visual Molecular Dynamics (VMD, version 1.9.3) software [65].

## 4. Conclusions

We have conducted a comprehensive investigation into the stability and electronic properties of mixed rare-earth tri-metallofullerenes, specifically Y_x_Dy_3−x_@C_80_ (x = 1 or 2), through a combined experimental and theoretical study. Our findings indicate that the chemical oxidation of the arc-discharge produced soot can result in the formation of cations, namely $Y_{2}Dy@C_{80}^{+}$ and ${YDy}_{2}@C_{80}^{+}$ . Through DFT calculations, we have determined that the tri-metallofullerenes Y_x_Dy_3−x_@C_80_ (x = 1 or 2) possess a low oxidation potential, significantly lower than that of the other fullerenes like C_60_ and C_70_. This low oxidation potential is attributed to the relatively high energy level of the singly occupied orbital alpha HOMO. Furthermore, the oxidized species exhibit a large HOMO-LUMO gap similar to that of Y_x_Dy_3−x_N@C_80_, highlighting their high chemical stability. Theoretical studies have revealed the presence of a three-center two-electron metal–metal bond at the center of $Y_{2}Dy@C_{80}^{+}$ and ${YDy}_{2}@C_{80}^{+}$ . This unique multi-center bond helps alleviate the electrostatic repulsion between the metal ions, thus contributing to the overall stability of the cations. 

## Figures and Tables

**Table 1:** The calculated relative energy (kcal/mol) of Y_2_Dy@C_80_ with different fullerene cages. The spiral number of the fullerene cage is included.

Molecular Formula	Relative Energy
Y_2_Dy@I_h_-_31924_C_80_	0.00
Y_2_Dy@D_5h_-_31923_C_80_	12.04
Y_2_Dy@C_2v_-_31922_C_80_	17.13
Y_2_Dy@C_1_-_28325_C_80_	19.50
Y_2_Dy@C_2_-_29591_C_80_	24.54
Y_2_Dy@C_2v_-_31920_C_80_	25.58
Y_2_Dy@C_1_-_31876_C_80_	28.06
Y_2_Dy@C_2_-_28319_C_80_	29.20
Y_2_Dy@C_1_-_28324_C_80_	30.50

**Table 2:** The calculated relative energy (kcal/mol) of YDy_2_@C_80_ with different fullerene cages.

Molecular Formula	Relative Energy
YDy_2_@I_h_-_31924_C_80_	0.00
YDy_2_@D_5h_-_31923_C_80_	12.08
YDy_2_@C_2v_-_31922_C_80_	18.67
YDy_2_@C_1_-_28325_C_80_	23.05
YDy_2_@C_2_-_29591_C_80_	27.81
YDy_2_@C_2v_-_31920_C_80_	28.11
YDy_2_@C_1_-_31876_C_80_	29.76
YDy_2_@C_2_-_28319_C_80_	30.49
YDy_2_@C_1_-_28314_C_80_	32.32

**Table 3:** The calculated relative energy (kcal/mol) of Y_2_Dy@I_h_-C_80_ and Y_2_DyC_2_@C_78_ with different fullerene cages. The spiral number of the fullerene cage is included.

Molecular Formula	Relative Energy
Y_2_Dy@I_h_-C_80_	0.00
Y_2_DyC_2_@C_2_-_22010_C_78_	42.95
Y_2_DyC_2_@C_1_-_21975_C_78_	59.17
Y_2_DyC_2_@C_2v_-_24107_C_78_	59.25
Y_2_DyC_2_@C_2v_-_24088_C_78_	71.57
Y_2_DyC_2_@D_3h_-_24109_C_78_	90.81

**Table 4:** The calculated relative energy (kcal/mol) of YDy_2_@I_h_-C_80_ and YDy_2_C_2_@C_78_ with different fullerene cages. The spiral number of the fullerene cage is included.

Molecular Formula	Relative Energy
YDy_2_@I_h_-C_80_	0.00
YDy_2_C_2_@C_2_-_22010_C_78_	49.10
YDy_2_C_2_@C_2v_-_24107_C_78_	63.04
YDy_2_C_2_@C_1_-_21975_C_78_	65.24
YDy_2_C_2_@C_2v_-_24088_C_78_	78.04
YDy_2_C_2_@D_3h_-_24109_C_78_	95.57

## Data Availability

All data reported in this study are available upon request by contact with the corresponding author. The data are not publicly available due to technical limitations (with a total size of a few GB).
